# Editorial: Updates on the complement system in kidney diseases

**DOI:** 10.3389/fimmu.2023.1196487

**Published:** 2023-04-06

**Authors:** Roberta Bulla, Mihály Józsi

**Affiliations:** ^1^ Department of Life Sciences, University of Trieste, Trieste, Italy; ^2^ Department of Immunology, ELTE Eötvös Loránd University, Budapest, Hungary; ^3^ MTA-ELTE Complement Research Group, Eötvös Loránd Research Network (ELKH), at the Department of Immunology, ELTE Eötvös Loránd University, Budapest, Hungary

**Keywords:** COVID-19, atypical hemolytic uremic syndrome (aHUS), complement system, Factor H (FH), factor H-related protein (FHR), kidney injury, extracellular matrix (ECM), C3 glomerulopathy

The complement system is an ancient arm of the immune system. As such, it is involved in various physiological processes, including protection against pathogenic microbes and tumor cells, disposal of waste material such as dead cells and cell debris, and removal of immune complexes. In addition, several other “non-canonical” complement functions have been recognized, including roles in the activation of various cells, developmental processes, and synaptic pruning. Complement activation is potentially deleterious to the host if proceeds unchecked; thus, disturbance in the fine balance between its activation and inhibition can lead to various pathologies. The kidney is one of the organs involved in several complement-mediated diseases. This Research Topic features 13 papers covering various aspects of the role of the complement system in kidney diseases.

## Role of the complement system in atypical hemolytic uremic syndrome

Atypical hemolytic uremic syndrome (aHUS) is a thrombotic microangiopathy (TMA) with complement abnormalities as predisposing factors. Pollack et al. reports a novel homozygous mutation in the central complement component C3, a deletion of four amino acids in the TED domain of the molecule. In the index patient, very low C3 levels were detected, indicating complement consumption due to overactivation. This is supported by molecular modeling, which showed that the deletion affects the interface between C3b and factor H (FH), a critical regulator of complement activation. In addition to C3 mutants, factor B (FB) variants can result in complement overactivation. Aradottir et al. studied three FB missense variants, two identified in aHUS patients and one in a patient with membranoproliferative glomerulonephritis. One of the FB mutants, D371G proved a gain-of-function variant, showed increased binding to C3b, causing enhanced formation of C3 convertase enzymes and thus excessive complement activation in assays using host cells. Importantly, the authors demonstrated that by applying danicopan, an inhibitor of factor D, the enzyme that cleaves FB when bound to C3b and thus generates the C3bBb convertase, efficiently blocks FB cleavage and complement overactivation also in the case of the gain-of-function FB mutant.

Factor I is the key enzyme cleaving and thus inactivating C3b in the presence of cofactors. Saleem et al. report a patient with membranous nephropathy who underwent kidney transplantation and developed aHUS after the transplantation. Genetic analysis of the patient revealed the I357M mutation in factor I in heterozygosis. While factor I antigenic levels were normal in the patient, this variant showed reduced expression in 293T cells and, more importantly, reduced activity in the presence of the soluble regulator FH as cofactor, while its activity in the presence of complement receptor type 1 and membrane-cofactor protein (i.e., membrane-bound cofactors) was not affected. These data identify the I357M factor I variant as a risk factor for developing post-transplant aHUS.

FH is the main inhibitor of the alternative pathway (AP) of complement in body fluids and recognizes and binds to host cells *via* sialic acid and glycosaminoglycans and inhibits the AP on these surfaces as well. Five FH-related proteins arose through gene duplications from the FH-coding *CFH* gene, namely FHR-1 to FHR-5, encoded by five *CFHR* genes. In the FHRs the domains responsible for the complement regulatory activity of FH are not conserved and they may compete with FH for binding to certain ligands and surfaces. Due to their highly homologous sequences, the genomic region containing the *CFH* and the *CFHR* genes in proximity is prone to rearrangements, deletions and duplications of exons or whole genes. Such rearrangements and single-nucleotide polymorphisms affect the risk of several diseases, including that of aHUS and C3 glomerulopathy (C3G). Piras et al. describe a detailed analysis of this gene cluster in a large cohort of patients and identified various hybrid genes, including previously unreported ones, whole gene duplications or internal duplications in *CFH*. They found that such alterations are relatively frequent in primary aHUS while occur rarely in secondary aHUS forms. This work highlight the association of genomic rearrangements in the *CFH-CFHR* gene cluster with aHUS, and the complexity of these genetic factors that are often difficult to identify.

## Role of the complement system in other renal diseases

Yoshida and Nishi in their mini review discussed the involvement of the complement system in TMA, a pathological condition caused by the formation of microvascular thrombi that leads to thrombocytopenia, microangiopathic hemolytic anemia, and organ damage. Kidney glomerular capillary thrombosis is mediated by complement dysregulation or complement overactivation. To understand how these vascular components interact will be fundamental for the creation of therapeutic strategies.

Complement activation contributes to the pathogenesis of acute kidney injury (AKI) in transplant patients. AKI is characterized by a rapid loss of renal function and is still associated to a high morbidity and mortality. The most common causes of AKI include renal ischemia-reperfusion injury, sepsis, and exogenous nephrotoxins such as drugs. AKI predisposes to the future development of chronic kidney disease (CKD) and subsequently to end-stage chronic renal disease. Currently, a specific treatment to arrest or attenuate progression in CKD is lacking. Franzin et al. reviewed recent findings on the role of complement in AKI-to-CKD transition. They also address how and when complement inhibitors might be used to prevent AKI and CKD progression improving graft function.

## Renal damage and infections

The kidney is the second most common organ affected by COVID-19.

Pfister et al. analysed kidney biopsies with acute kidney failure for complement factors C1q, MASP-2, C3c, C3d, C4d and C5b-9. The classical pathway and C3 cleavage products were strongly detected. The membrane attack complex C5b-9 was also found deposited in peritubular capillaries, renal arterioles, and tubular basement membrane. They concluded that specific complement inhibition might be a promising therapeutic strategy in COVID-19 patients.

Bouwmeester et al. described the association between both Pfizer/BioNTech’s (BNT162b2) mRNA-based and AstraZeneca’s (ChAdOx1 nCoV-19) adenoviral-based COVID-19 vaccines and aHUS in the Dutch population. They identified COVID-19 vaccination as a potential trigger for aHUS onset or relapse in pediatric and adult patients who were not treated with C5 inhibition. Therefore, aHUS should be included in the differential diagnosis of patients with vaccine-induced thrombocytopenia, especially if co-occurring with mechanical hemolytic anemia and severe acute kidney injury, but in the absence of major neurological complications.

van Beek et al. demonstrated that renal failure was associated with the decrease of FH in the plasma as a consequence of the severity of meningococcal disease. In their study the serum levels of FH and all FHRs had been measured from a cohort of pediatric meningococcal disease patients during the acute stage of disease in relation to *Neisseria meningitidis* serogroup, diagnosis and severity parameters, and compared these with levels during convalescence in surviving patients. The authors concluded that plasma concentrations of all FH family proteins were greatly decreased during the acute phase of meningococcal disease. However, predominantly low FH plasma concentrations were associated with the severity of meningococcal disease and renal failure.

## Role of extracellular matrix and heparan sulfate proteoglycans in the regulation of complement activation

Components of the extracellular matrix (ECM), when exposed to body fluids may promote local complement activation and inflammation. Binding of soluble complement inhibitors to the ECM, such as factor H (FH), is important to prevent excessive complement activation locally. Pathologic complement activation at the glomerular basement membrane is implicated in renal diseases.

Papp et al. demonstrated that the FH-Related Proteins FHR-1 and FHR-5 can interact with the ECM and reduce FH regulatory activity and enhance complement activation. They showed that FHR-1 and FHR-5 bind to ECM elements like does FH, and that both FHRs competitively limit binding of FH, thus reducing complement regulation. By this activity, FHRs may influence the pathogenic and inflammatory conditions in kidney, eye, and joint diseases.

Another study by Loeven et al. demonstrated that the relative balance of FH and FHR-1/FHR-5 in the glomerular glycocalyx is affected by HS-mediated ligand selectivity, which alters complement AP regulation in this milieu. These findings offer novel insights into the pathogenesis of C3G and imply that genetic testing on C3G cohorts can identify patients who have mutations in HS proteoglycan production genes that result in a “permissive” milieu that promotes FHR over FH binding. In turn, this imbalance fosters complement dysregulation either directly or indirectly through additional triggering events. These results also point to a potential C3G therapy in which FHR-1 and FHR-5 are scavenged by short 2-O-desulfated heparin oligosaccharides, changing their affinity for the glomerular glycocalyx.

## New experimental models for the diagnosis and treatment of kidney diseases

Overactivation of the AP of complement in the fluid phase and on the surface of the glomerular endothelial glycomatrix is the underlying cause of C3G. Pisarenka et al. developed an *in vitro* model of AP activation and regulation on a glycomatrix surface using an extracellular matrix substitute (MaxGel) for the reconstitution of AP C3 convertase. This ECM-based model of C3G offers a replicable method by which to evaluate the variable activity of the complement system in the context of disease.

Gaykema et al. demonstrated that CD55 over-expressing human induced pluripotent stem cells (iPSCs) and their derived kidney organoids are less susceptible to complement activation *in vitro*, providing evidence for the use of CD55 genetic manipulation to improve transplant outcomes of allogenic iPSC-derived tissues.

## Conclusions

In summary, this Research Topic highlights novel aspects of the involvement of the complement system in kidney disease, including functional characterization of disease-associated mutations, mechanisms of activation and regulation, and association with infections. In addition, new experimental models are presented to aid improved diagnostics and research, and the potential of complement inhibition to prevent or mitigate kidney damage is discussed.

## Author contributions

Both authors have made a substantial, direct, and intellectual contribution to the work and approved it for publication.

